# How to make a sex chromosome

**DOI:** 10.1038/ncomms12087

**Published:** 2016-07-04

**Authors:** Alison E. Wright, Rebecca Dean, Fabian Zimmer, Judith E. Mank

**Affiliations:** 1 Department of Genetics, Evolution and Environment University College London, London WC1E 6BT UK

## Abstract

Sex chromosomes can evolve once recombination is halted between a homologous pair of chromosomes. Owing to detailed studies using key model systems, we have a nuanced understanding and a rich review literature of what happens to sex chromosomes once recombination is arrested. However, three broad questions remain unanswered. First, why do sex chromosomes stop recombining in the first place? Second, how is recombination halted? Finally, why does the spread of recombination suppression, and therefore the rate of sex chromosome divergence, vary so substantially across clades? In this review, we consider each of these three questions in turn to address fundamental questions in the field, summarize our current understanding, and highlight important areas for future work.

## Abstract (web summary)

Sex chromosome evolution begins when recombination between a homologous pair of chromosomes is halted. Here, Wright *et al.* review our current understanding of the causes and mechanisms of recombination suppression between incipient sex chromosomes and suggest future directions for the field.

## Graphical abstract

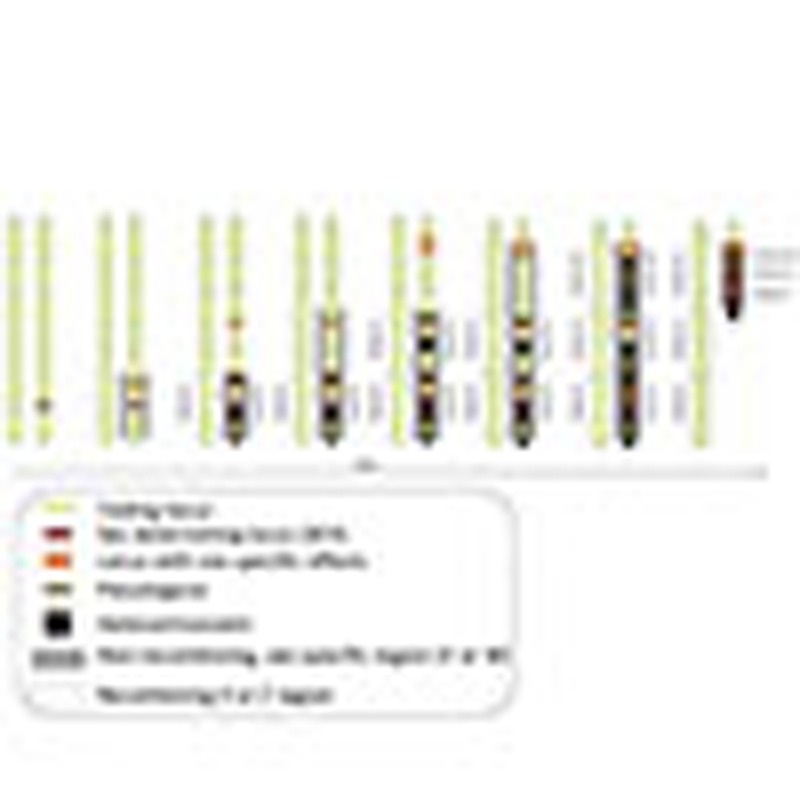

Sex chromosomes have evolved independently many times throughout the eukaryotes, and represent a remarkable case of genomic convergence, as unrelated sex chromosomes share many properties across distant taxa^1^,^2^,^3^. Sex chromosomes evolve after recombination is halted between a homologous pair of chromosomes^4^,^5^, leading to a cascade of non-adaptive and adaptive processes that produce distinct differences between the X and Y (or Z and W) chromosomes.

Owing to detailed studies in *Drosophila*^6^,^7^,^8^ and mammals^9^,^10^,^11^, we have a nuanced understanding of the consequences of arrested recombination^1^,^4^,^7^,^8^. The non-recombining Y and W chromosomes become highly heterochromatic (see Box 1 for a glossary) and experience profound levels of gene loss even as the X and Z chromosomes remain functional^1^,^12^,^13^,^14^. Sex chromosomes have been the focus of intense study and are an important model for understanding the consequences of recombination suppression^12^,^15^. It is clear that the loss of recombination triggers a host of evolutionary processes, including Muller's Ratchet, background selection and genetic hitchhiking, reviewed in ref. 16, that lead to the loss of gene activity and pseudogenization (detailed in Box 2). This work makes very clear the evolutionary consequences of halting recombination between the sex chromosomes.

Why recombination is suppressed in the first place is less clear, as the chromosomes that determine sex in many organisms with genetic sex determination never progress to heteromorphic sex chromosomes. For example, a single missense single nucleotide polymorphism in the coding region of the *Amhr2* locus appears to control sex in the tiger pufferfish (*Takifugu rupripes*)^17^, but recombination is not restricted around this sex-determining gene and there is no evidence of divergence beyond this single nucleotide between the proto-X or proto-Y. Similarly, despite considerable age, the sex chromosomes in many clades (including ratite birds^18^,^19^, pythons^20^ and European tree frogs^21^) have failed to develop substantial heteromorphism, and remain largely identical.

These observations indicate that recombination suppression and sex chromosome divergence are not inevitable consequences of genetic sex determination, leading to three questions at the heart of sex chromosomes evolution. First, why do sex chromosomes stop recombining? Second, how is recombination suppression achieved? Third, why does the spread of recombination suppression, and therefore the rate of sex chromosome divergence, vary so substantially across clades?

The implications of these questions go far beyond sex chromosome research *per se*. Recombination rate has long been known to be a critical factor in the ability of a genomic region to respond to selection. Dobzhansky and colleagues^22^,^23^,^24^,^25^ noted that halting recombination can permanently link co-adapted gene complexes (recently renamed supergenes) within populations. These supergenes are then transmitted as a unit, allowing for complex adaptions spanning multiple loci. More recently, the importance of recombination has resurfaced in evolutionary biology with several key examples in a range of species implicating recombination suppression as a crucial component of complex phenotypic adaptation^26^,^27^,^28^,^29^ and speciation^30^. The study of sex chromosomes therefore offers a route to understand the interplay between recombination, selective forces and adaptation, with broad implications across multiple fields of evolutionary genetics.

## Why do sex chromosomes stop recombining

### The sexual conflict model of sex chromosome evolution

The most commonly accepted theory of sex chromosome evolution^14^,^31^,^32^ predicts that recombination will be selected against in the region between a sex-determining gene and a nearby gene with sex-specific effects (Box 2). This theory was based in part on early studies of colouration genetics in the guppy, *Poecilia reticulata*^33^, which demonstrated that many genes underlying male colouration are Y-linked. Colouration genes are sexually antagonistic—they benefit males through increased reproductive success but are detrimental to both sexes due to increased predation. For males, the benefits of increased mating opportunities outweigh the costs when predation pressures are not too high. In contrast, females gain no benefit from displaying bright colours to offset increased predation, as males are not attracted to ornamented females. Linkage between the allele that confers maleness at the sex determining locus and the allele for bright coloration at a nearby locus creates a male supergene—the allele determining maleness is always co-inherited with the linked allele, which confers a fitness benefit in males. The linkage of these alleles also resolves sexual conflict over colour between males and females, as the colouration allele would no longer be present, and therefore selected against, in females.

Although the sexual conflict model of sex chromosome evolution remains widely accepted, the evidence for or against it is remarkably slim. Non-adaptive alternatives have been suggested as well^34^,^35^, but also lack definitive evidence. Clear empirical evidence to support the sexual conflict theory of sex chromosome evolution is limited in part because the main model species for empirical studies of sex chromosome evolution exhibit highly derived X and Y chromosomes, requiring substantial extrapolation to infer the initial stages of divergence.

Importantly, it can be difficult in ancient systems to differentiate cause from consequence. For example, the gene content of the Y chromosome has been interpreted as supporting the role of sexual conflict in sex chromosome evolution. The Y chromosome in mammals^36^ and *Drosophila*^37^,^38^, as well as the analogous W chromosome in birds^39^, contains loci essential to sex-specific fitness, which might have been sexually antagonistic before they became sex-limited (linked to the Y or W chromosome). However, although sexual conflict over these loci could have catalyzed sex chromosome divergence through selection for recombination suppression (supporting the sexual conflict model), these genes could just as easily have relocated after recombination halted^40^. In support of this latter explanation, there is evidence of strong selection for the relocation of male-benefit gene duplicates to the Y chromosome in *Drosophila*^40^. Alternatively, these genes may have developed sex-specific functions after the sex chromosomes diverged, as there is also evidence that loci on sex chromosomes adapt to their sex-specific environment once recombination ceases^41^. Y-linked loci would therefore be more likely to adopt male-specific functions after recombination with the X chromosome is halted, but these functions would not drive recombination suppression itself.

Evidence from sex chromosome systems at earlier stages of divergence is therefore key to understanding why sex chromosomes evolve, and there are a wealth of systems with early stage sex chromosomes including *Anolis* lizards^42^,^43^, anurans^21^,^44^,^45^, snakes^46^, fish^47^, many plants^48^,^49^,^50^,^51^, among numerous others^2^. However, although these systems have revealed several important characteristics of early stage sex chromosome evolution, the difficulty in identifying sexually antagonistic alleles at the molecular level has hampered direct empirical tests of the sexual conflict model. Indirect evidence for the sexual conflict model comes from the three-spine stickleback (*Gasterosteus aculeatus*), where a neo-sex chromosome fusion in the Sea of Japan population may have been driven, at least in part, by sexual conflict^52^. However, recombination suppression has not spread across the added region, suggesting that linkage between the sexually antagonistic locus and the sex determining locus may not explain the fusion event^53^. Similarly, a sexually antagonistic colouration pattern has been mapped to the W chromosome in some cichlids^54^; however, given the dynamic and polygenic nature of sex determination in cichlids^55^, it is not clear whether W-linkage predates sex chromosome evolution or that linkage of the coloration locus to the sex determining gene led to recombination suppression.

### Transitions from hermaphroditism to sex chromosomes

The theory of sex chromosome evolution articulated above assumes that the separation of the sexes, called gonochorism in animals and dioecy in plants, predates the evolution of sex chromosomes. Because of this assumption, the theory is in many ways more applicable to animals, which are more often gonochoristic. Dioecy is rare in plants, which restricts the evolution of sex chromosomes to fewer taxa. In flowering plants (angiosperms), only 5–6% of all species have separate male and female genders^56^. Of the dioecious angiosperms, only a small number have been shown to possess sex chromosomes of which roughly half are homomorphic^56^,^57^. However, without detailed genetic analysis, homomorphic sex chromosomes are difficult to identify. As a result, there may be many cryptic homomorphic species where the sex chromosomes are karyotypically indistinguishable and just waiting to be discovered.

In plants and other systems where sex chromosomes are associated with transitions from hermaphroditism to separate sexes, sex chromosome formation may take a slightly different route than in species with ancestral separate sexes. In this case, the dominant model^58^ predicts that separate male- and female-sterile mutations on the same chromosome cause the shift from hermaphroditism to dioecy through an intermediate phase of gynodioecy. Once these mutations have occurred and reached sufficient frequency in the population, recombination suppression between them prevents reversal back to hermaphroditism, leading to the evolution of sex chromosomes. Recent evidence from wild strawberry^59^ and papaya^49^,^60^ has provided insight into these early stages of sex chromosome evolution in plants and the availability of genomic tools will help us understand how recombination is suppressed between feminizing and masculinizing alleles.

## How is recombination halted between the sex chromosomes

Regardless of why sex chromosomes originate, the process of sex chromosome evolution necessitates halting recombination between the nascent X and Y in males, or Z and W in females. Therefore, sex chromosome evolution at the most basic level requires sex-specific recombination patterns on the sex chromosomes. Recombination varies substantially in males and females, both in frequency and in specific hotspots, referred to as heterochiasmy. An extreme example of this is achiasmy, where recombination only occurs in one sex^61^.

Achiasmy may either precede or follow emergence of a nascent sex determining locus^62^,^63^, and in either case, can accelerate sex chromosome divergence. For example, in an achiasmate species, the emergence of a nascent sex determining factor leads to instantaneous recombination suppression along the entire length of the sex chromosomes. Similarly, when achiasmy follows quickly after the emergence of a nascent sex determining factor, recombination suppression also occurs along the entire length of the sex chromosomes. Only when achiasmy evolves in systems with highly differentiated sex chromosomes would it not be expected to foster sex chromosome divergence. As a result, the sex chromosomes of achiasmate species tend to have a single heteromorphic stratum, as the emergence of a new sex determining allele causes the entire sex chromosome to start to diverge^64^.

**Figure 1:**
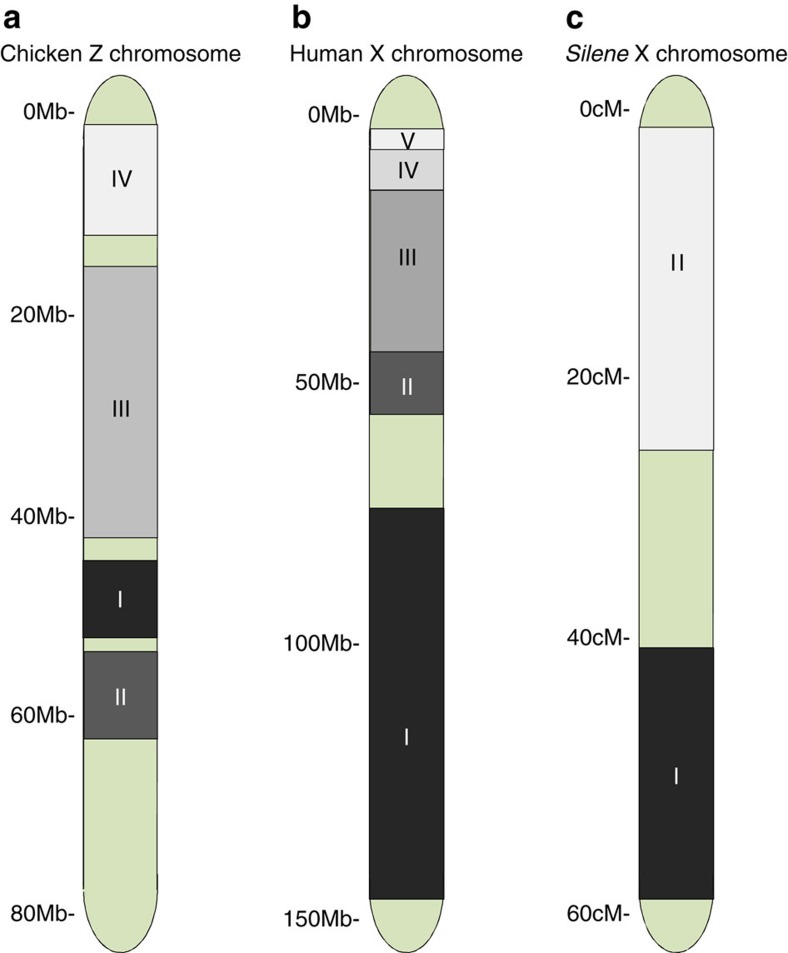
Sex chromosome strata. Many plants and animals show evidence of strata, spatial clusters of X-Y, or Z-W, orthologs with similar divergence estimates. These spatial clusters are consistent with inversion events instantaneously halting recombination for all the encompassed loci. As inversions are proposed to occur in a stepwise process, strata differ in the length of time over which recombination has been suppressed. Therefore, orthologs with the largest neutral sequence divergence reside in the oldest stratum (shown in black), whereas those with the greatest sequence similarity are located in the youngest stratum (shown in white). The chicken Z chromosome (**a**) is comprised of at least four strata, formed over 130 million years^68^ and the human X chromosome (**b**) is comprised of at least five strata^105^, although some recent analyses support six or more strata^106^,^107^. The *Silene* X and Y chromosomes (**c**) diverged more recently and there is evidence for two strata over 10 million years^66^. However, it is possible that orthology-based approaches underestimate the number of strata (regions unassigned to strata shown in green). For example, in highly degenerated regions, often all of the Y or W loci have decayed and no orthologs remain. In these cases, alternative methods have been used to identify additional strata^92^,^108^.

In species where both sexes recombine, some mechanism is needed to block recombination between the sex determining gene and nearby genes with sex-specific effects in the heterogametic sex. Chromosomal inversions spanning the sex determining locus and nearby sexually antagonistic loci are often assumed to halt recombination and therefore to drive sex chromosome divergence^65^. There is circumstantial evidence implicating inversions in sex chromosome evolution. For example, sex chromosomes in many animals and plants show evidence of strata, spatial clusters of X-Y or Z-W orthologs with similar divergence estimates (Fig. 1)^10^,^20^,^48^,^66^,^67^,^68^. These spatial clusters are consistent with inversion events instantaneously halting recombination for all the encompassed loci. However, reports from nascent sex chromosomes suggest that recombination suppression is initially heterogeneous across the sex chromosomes^53^,^69^,^70^, implying that recombination suppression evolves initially by another, uneven mechanism, inconsistent with large-scale inversions.

Recombination is dynamic and heterogeneous, and the rate of recombination varies extensively throughout the genome and between the sexes^63^,^71^. For species where both sexes recombine, local sex-specific recombination rates may be important initially in sex chromosome divergence, although the mechanism for sex-specific heterochiasmy is not yet known (Box 3). Importantly, regardless of the mechanism, once recombination has been halted in the heterogametic sex, selection to maintain gene order is abolished^72^ and inversions are less likely to be selected against. Relaxed selection against inversions suggests that inversions might follow recombination suppression. Therefore, it remains unclear whether inversions catalyze or are a consequence of halting recombination between sex chromosomes.

Recent work on recombination evolution has suggested that sequence characteristics, namely binding motifs and structural traits, can exhibit short-term evolutionary dynamics that can lead to rapid shifts in local recombination rates^73^,^74^,^75^. Although not present in all species^76^,^77^, when they are associated with recombination, rapid changes in these motifs lead to differences in recombination rates in specific genomic locations among closely related species^73^,^78^,^79^, and even among conspecific populations^71^,^74^,^80^. The role of structural modifications and binding motifs in sex chromosome evolution, as well as other genetic and epigenetic mechanisms (detailed in ref. 81), have yet to be explored, but these mechanisms offer plausible alternatives to inversions in driving recombination suppression.

## Why do sex chromosomes diverge at such different rates

### Homomorphic sex chromosomes are curiously common

Many organisms with genetic sex determination lack heteromorphic sex chromosomes, indicating that the non-recombining region has not spread significantly beyond the sex determining locus. Examples of animal systems with homomorphic sex chromosomes include the pufferfish^17^, ratite birds^18^,^19^, pythons^20^ and European tree frogs^21^. Also, many dioecious species of flowering plants possess homomorphic sex chromosomes^82^. The reasons why sex chromosomes might remain largely undifferentiated are not well understood, but here we suggest five possible explanations.

### Age

First, some homomorphic sex chromosomes are young and may be in the early stages of degeneration, for example in papaya^49^,^60^. However, in many species, the sex chromosomes are old and yet have not degenerated, such as in European tree frogs^21^, pythons^20^ and ratite birds^19^. Thus, we must conclude that age is not always an accurate predictor of the relative size of the non-recombining region, and therefore of overall sex chromosome divergence.

### Relative length of haploid phase

Some organisms have a long haploid phase, resulting in strong haploid purifying selection acting to maintain gene activity on the Y chromosome^70^,^83^,^84^. In species where haploid selection is more limited, many genes on the Y or W chromosome are sheltered in the diploid phase by the copy on the X or Z chromosome, and purifying selection may only act on dosage sensitive genes to maintain sufficient gene activity. Therefore, we might expect slower W or Y degeneration in species where haploid selection is more pervasive, such as algae and plants, compared with species where it is less widespread, such as animals. Similarly, some animals have a much reduced haploid phase in females compared to males, and this might retard W chromosome degeneration compared to that of Y chromosomes^63^.

### Sex chromosome dosage compensation

**Figure 2:**
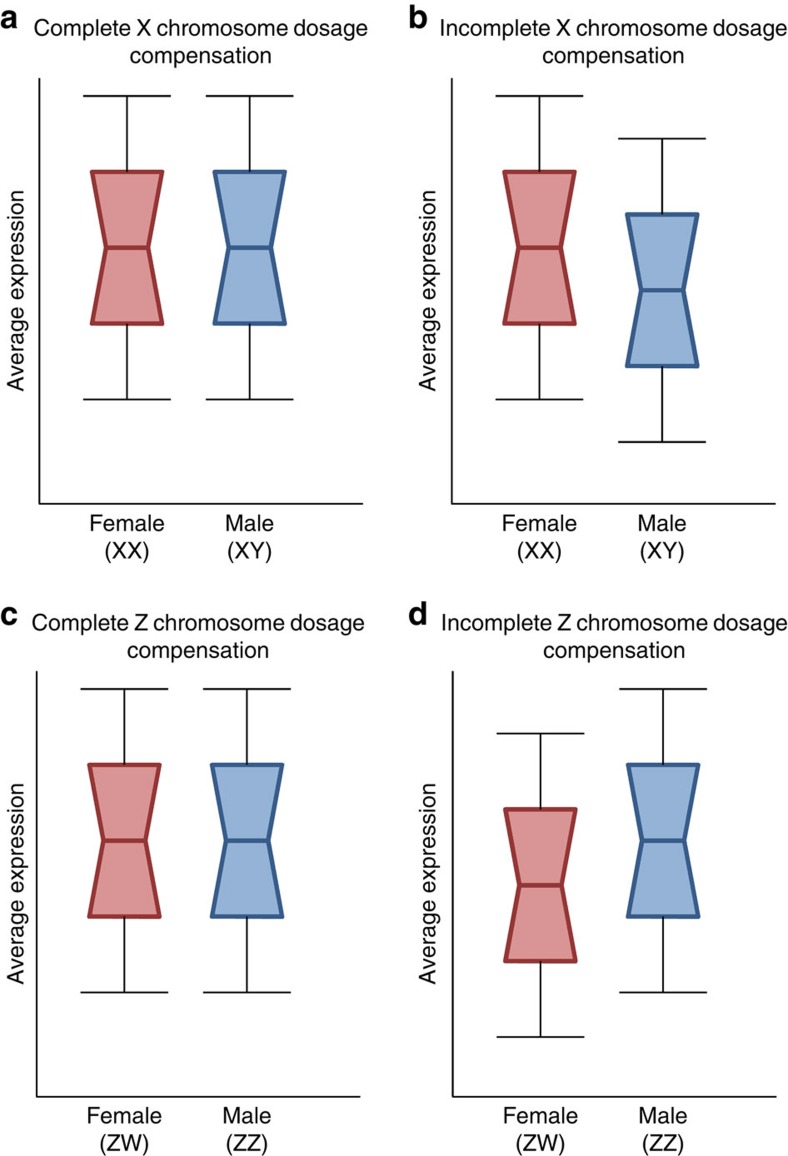
Cartoon illustration of sex chromosome dosage compensation. The decay of Y and W chromosome gene content leads to differences in gene dose (the number of gene copies) between the sexes. In male heterogamety (**a**,**b**) males have one half of the dose of all X-linked genes lost from the Y chromosome. In some cases, this difference in gene dose has led to the evolution of complete sex chromosome dosage compensation (**a**), where a mechanism acts across the chromosome to balance out the differences in gene dose, and as a consequence, the average expression for X-linked genes is equal in males and females. In many other cases (**b**), only some genes on the X are compensated, and the average expression from the X chromosome is less in males than females. In female heterogamety (**c**,**d**) females have one half of the dose of all Z-linked genes lost from the W chromosome. In some cases, this difference in gene dose has led to the evolution of complete sex chromosome dosage compensation (**c**), but in many other cases (**d**), only some genes on the Z are compensated, and the average expression from the Z chromosome is less in females than males.

After recombination has been halted between the sex chromosomes, the non-recombining Y or W chromosome decays^85^. A consequence of this degeneration is that gene dose is reduced on the X and Z chromosomes relative to the autosomes in the heterogametic sex. This imbalance in gene expression is often thought to be detrimental, and upsets the biochemical stoichiometry of interacting gene products. These deleterious effects were hypothesized to drive the evolution of dosage compensation mechanisms in order to restore ancestral diploid expression levels^86^. The extent of dosage compensation varies significantly across taxa^87^, and although some species exhibit complete sex chromosome dosage compensation, many more show incomplete compensation (reviewed in refs 87, 88, shown in Fig. 2). The factors underlying this variation are not at all clear and may include sexual conflict over optimal gene expression^89^, as well as variation in effective population size and male-biased mutation rates.

Much of our understanding of Y chromosome decay comes from the neo-sex chromosomes in *Drosophila* and the X-added region of the eutherians. In both these cases, an existing system of complete dosage compensation quickly spread onto the expanded X chromosome^90^,^91^. The spread of an existing mechanism of dosage compensation onto a neo-sex chromosome would reduce the power of purifying selection to maintain gene activity on dosage sensitive neo-Y orthologs, in turn leading to an acceleration of neo-Y chromosome decay.

The slow rate of gene decay recently observed on the W chromosome in birds^92^ provides a stark contrast to the *Drosophila* and eutherian Y, and it was recently suggested that this difference is largely due to the opposing effects of male-biased mutation on Y and W chromosomes^1^,^93^. However, birds have only incomplete sex chromosome dosage compensation^87^, raising questions about the generality of the lessons from the *Drosophila* neo-sex chromosomes and the eutherian X-added region, as well as suggesting that the dichotomy between *Drosophila* and eutherians versus birds might not be heterogamety (XY versus ZW), but rather complete versus incomplete dosage compensation. Recent work in sticklebacks, a male heterogametic system with incomplete dosage compensation, indicates that purifying selection remains strong on dosage sensitive Y genes^94^. Therefore it may be that in systems with incomplete dosage compensation, Y or W degeneration might be retarded through purifying selection acting on dosage sensitive genes, and that dosage compensation status may be a major factor underlying differences in sex chromosome degeneration rates.

### Sex reversal

Sex reversal, discordance between an individual's phenotypic and genotypic sex, may be important in recombination suppression and sex chromosome evolution. In many ectotherm vertebrates, such as amphibians^95^,^96^ and teleost fish^97^, sex reversal results in reproductively viable individuals. Interestingly, because recombination patterns typically follow phenotypic but not genotypic sex, recombination can occur along the full length of the sex chromosomes in individuals with phenotypes that do not match their sex chromosome complement. Even when at very low frequency in the population, sex reversal can prevent sex chromosome divergence and lead to very old homomorphic sex chromosomes^98^, as has been shown in frogs^21^,^99^,^100^.

### Sexual conflict

Sexually antagonistic alleles are central to the sexual conflict model of sex chromosome evolution^32^, and systems with more sexual conflict experience more rapid expansion of the non-recombining region simply because more loci within the genome, and by extension proximate to the sex determining locus, carry sexually antagonistic alleles^101^. Heteromorphic sex chromosomes might be therefore expected to occur more often in lineages with high levels of sexual conflict and/or sexual dimorphism. However, sexual conflict might also trigger turnover of sex chromosomes^102^,^103^, thereby restarting the process of sex chromosome divergence. It is therefore unclear whether we should expect a direct relationship between the degree of sexual conflict and the size of the non-recombining region.

## Conclusion

Three major questions regarding the evolution of sex chromosomes remain unanswered. To answer them, it will be important to move well beyond the main model systems, and develop new study systems at earlier stages of sex chromosome divergence.

Does sexual conflict drive sex chromosome evolution? The role of sexual conflict in driving sex chromosome evolution, although widely accepted, remains fundamentally unknown, largely due to difficulties in identifying sexually antagonistic alleles directly. In order to answer this question, it is important that we develop new study systems with far younger sex chromosomes. Crucially, these study systems will also need to have some phenotypic trait or traits that are known to be sexually antagonistic, with known underlying genetic architecture. Alternatively, experimental evolution of sexual conflict may prove useful in studying changes in sex-specific recombination rates.

How is recombination suppressed between the sex chromosomes? The mechanisms underlying recombination suppression are still largely unknown. Inversions are often assumed to facilitate sex chromosome divergence through recombination suppression, but this assumption is contradicted by the heterogeneity in divergence observed in young sex chromosome systems. Moreover, in old sex chromosome systems, it may be impossible to determine whether inversions catalyze sex chromosome evolution or are a consequence of recombination suppression achieved through other means. This difficulty in differentiating cause and effect again suggests that study systems with nascent sex chromosomes are crucial for understanding the cause of recombination suppression.

Why do rates of sex chromosome divergence vary so significantly across groups? Preliminary evidence suggests that the presence or absence of complete dosage compensation, the relative length of the haploid phase in the life cycle, and the prevalence and fertility of sex reversed individuals might be the largest predictors of the power of purifying selection to maintain gene activity on the sex-limited chromosome, and therefore the rate of gene loss once recombination is halted. The pervasiveness of sexual conflict throughout the genome may also be important. Untangling the role of these different characteristics in explaining the rate of sex chromosome divergence will require very large-scale comparative datasets and phylogenetic methods. Work in this direction has started^104^, but much more work is needed.

## Additional information

**How to cite this article:** Wright, A.E. *et al.* How to make a sex chromosome. *Nat. Commun.* 7:12087 doi: 10.1038/ncomms12087 (2016).

## Glossary.

Achiasmate: Complete suppression of recombination in one sex, typically the heterogametic sex. Observed in *Drosophila* and Lepidoptera, among others.

Dioecy: Botanical term for separate male and female flowers in different individuals. Similar to gonochorism in animals.

Dosage compensation: Gene regulation mechanism on the sex chromosomes to correct for differences in gene dose for the X or Z chromosome between the homogametic and heterogametic sexes (Fig. 2). A consequence of dosage compensation is that gene dose is equalized between males and females.

Female heterogamety: Sex chromosome type where females have a ZW karyotype, and males a ZZ karyotype. Present in birds, lepidoptera, snakes and anguillid eels.

Gonochorism: Animal term for separate sexes. Similar to dioecy in plants.

Gynodioecy: Male sterile individuals and hermaphrodites in the same population.

Heterochiasmy: Sex-specific variation in recombination rates.

Heteromorphic sex chromosomes: Sex chromosomes that are karyotypically highly distinct from one another. In these cases, the X and Y (or Z and W) chromosomes show major differences in size and gene content.

Homomorphic sex chromosomes: Where the X and Y (or Z and W) chromosomes exhibit few differences from each other in size and gene content, and are difficult or impossible to distinguish from karyotype data alone.

Hermaphrodite: Male and female reproductive organs in the same individual.

Male heterogamety: Type of sex chromosome system where females karyotype is XX, and male karyotype is XY. Observed in mammals, *Drosophila*, salmon as well as many beetles.

Pseudo-autosomal region: Regions where recombination persists between the X and Y (or Z and W) sex chromosomes. These regions, identical in both sexes, aid chromosome pairing during meiosis and ensure proper segregation.

Pseudogene: DNA sequences that once encoded protein sequences, but which are no longer transcribed into messenger RNA (mRNA) in a way that translates to functional protein.

Stratum: Region on the sex chromosomes where recombination has been suppressed. Strata can be identified by spatial clusters of X-Y or Z-W orthologs with similar divergence estimates.

## Theoretical model of sex chromosome differentiation.

Sex chromosomes evolve from autosomes, initially with the acquisistion of a sex determining locus (**a**). Emergence of sexually antagonistic alleles at loci in close proximity to the sex determining locus selects for recombination suppression between the X and Y or Z and W chromosome (**b**), resulting in Stratum I, which is increasingly heterochromatinized. Once recombination is halted on the Y or W chromosome genes without sex-specific benefits are often pseudogenized. The non-recombining region can expand with the acquisition of additional sexually antagonistic alleles and further recombination suppression, leading to additional strata—spatial clusters of X-Y or Z-W orthologs with similar divergence estimates, observed in mammals^9^, birds^39^,^109^, fish 67,94 and plants^48^,^66^, which also undergo loss of gene function and heterochromatinization (**d**–**g**). The lack of recombination leads to accumulation of repetitive DNA, which can lead to a short-term increase in the size of the Y or W, but which typically results in large-scale deletions, a large reduction in physical size of the sex-limited chromosome, and highly heteromorphic sex chromosomes (**h**)^7^,^65^.

Sex chromosomes may emerge in a somewhat different way in species where one sex or the other lack recombination at all. Referred to as achiasmy, this occurs in a range of species, most notably *Drosophila*^110^ and Lepidoptera^111^,^112^, but also within Hemiptera^113^, Heteroptera^114^,^115^,^116^ and Orthoptera^117^ and with restricted distributions in several other taxa^61^. In these cases, if achiasmy precedes the emergence of a nascent sex determining locus, linkage between two or more loci is not required for recombination to cease between the emergent sex chromosomes. The advent of a sex determining allele automatically makes the entire chromosome sex-limited and therefore non-recombining. In these cases, there are no discernible strata.

## Sex-specific recombination.

Recombination rates show substantial variation within the genome^73^,^77^,^118^,^119^, within species^71^,^74^,^80^ as well as across related species^73^,^78^,^79^. Importantly for sex chromosome evolution, there are often also differences between males and females in recombination rate, and sex differences in recombination rates are thought to occur in >75% of recombining species. In many cases, the magnitude of the difference can be very large^62^,^63^,^120^. In general, males tend to have lower rates of recombination than females during meiosis and this pattern is independent of male or female heterogamety^63^.

Sex-specific recombination rates, and in particular local sex-specific recombination cold-spots, may be important for initiating sex chromosome degeneration. Furthermore, sexual dimorphism in recombination could promote the spread of sexually antagonistic alleles, as low recombination in the sex that benefits from the sexually antagonistic genes keeps favourable sexually antagonistic combinations together^121^, which in turn could drive expansion of the non-recombining region and progressive sex chromosome evolution. Yet the evolutionary forces and molecular mechanisms driving sex-specific recombination are relatively unknown. Possible selective forces causes include stronger haploid selection in males than females^63^ and various forms of epistatic selection^62^. Understanding the mechanisms underlying recombination cessation, what causes inter- and intra-specific recombination rates, and whether achiasmate recombination is a cause or consequence of sex chromosome evolution will provide greater understanding of sex chromosome evolution.
